# The Link Between Adherence to Modern Myths About Sexual Aggression and Attachment Styles Among Young Physicians

**DOI:** 10.1192/j.eurpsy.2026.11793

**Published:** 2026-07-31

**Authors:** D. Triki, F. Guermazi, M. Abdelkafi, N. Regaieg, I. Feki, J. Masmoudi, I. Baati

**Affiliations:** Psychiatry A, Hedi Chaker university hospital, Sfax, Tunisia

## Abstract

**Introduction:**

Modern myths about sexual aggression (AMMSA) are pervasive false beliefs that blame victims, exonerate perpetrators, and minimize the severity of sexual violence. These myths represent a significant social and clinical problem, particularly among healthcare professionals who are often first-line responders for victims. Concurrently, adult attachment styles, which govern patterns of relations, fundamentally shape how individuals perceive interpersonal interactions and intimacy.

**Objectives:**

This study investigates the link between attachment styles (anxiety and avoidance) and adherence to modern myths about sexual aggression among a sample of young physicians.

**Methods:**

A cross-sectional, descriptive, and analytical study was conducted over two months (April-May 2025) among 153 interns and residents in Sfax-Tunisia. Participants completed an online questionnaire, which included a socio-demographic data sheet, the validated French version of Acceptance of Modern Myths about Sexual Aggression Scale (AMMSA), and the Experiences in Close Relationships scale-Short form (ECR-S) (attachment-related anxiety (6 items) avoidance (6 items)).

Data analysis was performed using SPSS.

**Results:**

The study population was predominantly female (74.5%) with a median age of 28. The median scores on the ECR-S were 24/42 [IIQ=20-26] for anxious attachment and 17/42 [IIQ=13-21] for avoidant attachment. The mean global AMMSA score was 102.05 ± 24.67. Statistical analysis revealed a significant positive correlation between the anxious attachment score and the AMMSA score (rho = 0.218; p=0.007). This indicates that higher levels of relational anxiety were associated with a stronger adherence to myths about sexual aggression. However, no significant correlation was found between avoidant attachment and the AMMSA score (rho=0.106; p=0.19).

**Conclusions:**

This study establishes a significant link between an anxious attachment style and greater adherence to modern myths about sexual aggression among young physicians. These results underscore the importance of considering psychological factors, such as attachment, in understanding the perpetuation of harmful beliefs.

**Disclosure of Interest:**

None Declared

